# Managing postoperative atrial fibrillation after open-heart surgery using transdermal β_1_ blockers

**DOI:** 10.1186/s13019-023-02227-z

**Published:** 2023-04-06

**Authors:** Kenji Yamamoto, Senri Miwa, Tomoyuki Yamada, Shuji Setozaki, Mamoru Hamuro, Shunji Kurokawa, Sakae Enomoto

**Affiliations:** 1 Department of Cardiovascular Surgery, Okamura Memorial Hospital, 293-1, Kakita Shimizu- cho, Sunto-gun, Shizuoka, Japan; 2 Department of Cardiovascular Surgery, Shiga General Hospital, 5-4-30, Moriyama, Moriyama- city, Shiga Japan; 3 Department of Cardiovascular Surgery, Shizuoka General Hospital, 4-27-1, Kitaandou, Aoi-ku, Shizuoka-city, Shizuoka Japan

**Keywords:** beta-blockers, Transdermal patch, Bisoprolol, Postoperative atrial fibrillation, Bisono tape

## Abstract

**Background:**

Postoperative atrial fibrillation (POAF) after open-heart surgery is a non-negligible complication. We aimed to describe the efficacy of a transdermal patch of bisoprolol for managing POAF and flutter in thoracic surgical procedures.

**Methods:**

We analyzed the data of 384 patients who underwent open-heart surgery at our hospital and received oral bisoprolol to prevent POAF. Among them, 65 patients (16.9%) also received a 4-mg transdermal patch of bisoprolol to control the heart rate due to POAF. We applied the bisoprolol transdermal patch when the heart rate was > 80 bpm and removed it at ≤ 60 bpm; an additional patch was applied when the heart rate was > 140 bpm. Heparin calcium injections were administered twice daily for anticoagulation between 2 and 6 days postoperatively.

**Results:**

The average number of prescriptions for transdermal patches of bisoprolol during hospitalization was 1.8 ± 1.1 (1–5). The median first prescription date was on postoperative day 2 (range: days 0–37). Sinus rhythm recovered within 24 h in 18 patients (27.7%). Eight patients (12.3%) were switched to continuous landiolol infusion because of persistent tachycardia. In three patients, the transdermal patch was removed owing to severe bradycardia. Fifteen patients experienced persistent atrial fibrillation and were treated with electrical cardioversion during hospitalization. We did not observe any serious complications that could be directly attributed to bisoprolol transdermal patch use.

**Conclusions:**

Single-use bisoprolol transdermal patch may help control the heart rate during the initial treatment of POAF after open-heart surgery.

## Background

Postoperative atrial fibrillation (POAF) after open-heart surgery is a non-negligible complication that can induce hemodynamic disruption and embolism, thereby complicating circulatory management and necessitating anticoagulation [1]. The 2014 American Association for Thoracic Surgery (AATS) guidelines recommend the continuous use of beta-blockers for the treatment of POAF [2], and intravenous landiolol is currently available and used for this purpose, exclusively in Japan [3]. In other countries, esmolol is the more commonly available β-blocker used for the treatment of POAF. However, in repeated sinus rhythm and atrial fibrillation (AF) cases, strict infusion and management may be complicated and economically challenging.

**Fig. 1:**
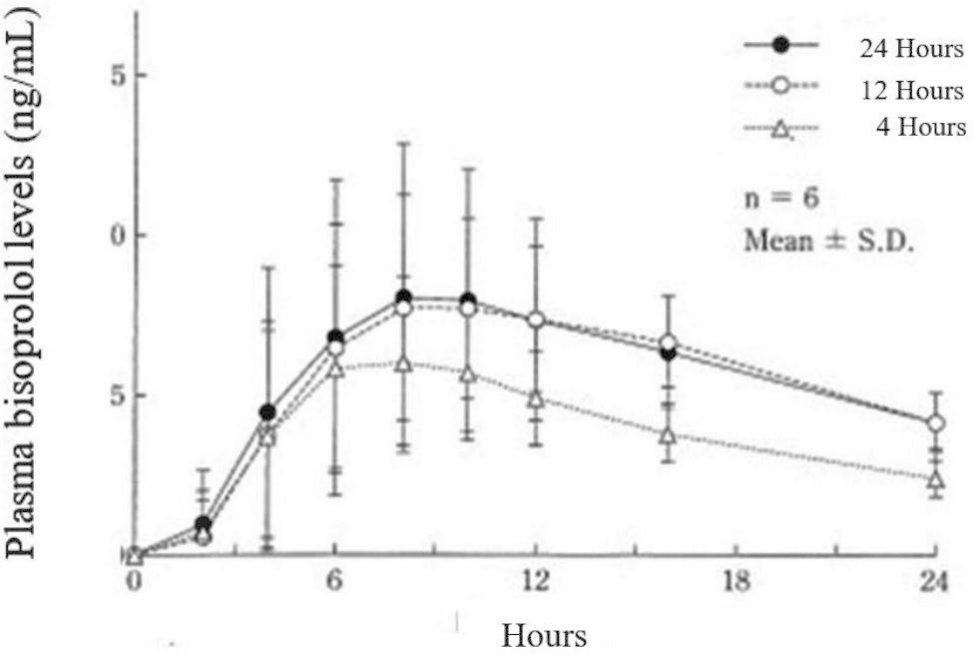
Midway peel test: changes in the plasma bisoprolol levels. This figure was reprinted from the article (Reference #4) with the author’s permission, and some parts were translated into English and reorganized. At approximately 4 h after the patch is removed after a single use, the plasma bisoprolol level gradually decreases

Recently, a transdermal beta-1 (β1) blocker (Bisono tape^®^, a transdermal patch of bisoprolol [TDPB]) has become available in Japan. It is relatively inexpensive, easy to use, and easy to remove. In single use, the blood concentration of transdermally absorbed bisoprolol rises more slowly and peaks lower compared to oral administration. Furthermore, if the patch is removed at 4 h after attachment, the blood concentration slowly decreases from 4 h after that time. Conversely, if the patch is retained for 12 h before being removed, the plasma bisoprolol level remains as if the patch was still attached for 24 h after removal [4] (Fig. 1). Previously, we investigated whether this TDPB can be safely used as an initial treatment for POAF, as this method has become increasingly popular in Japan [5]. According to recently emerging reports on TDPB from other facilities, TDPB is well tolerated and effective in patients with AF after noncardiac surgery [6]. The incidence of POAF in TDPB users is lower than that in oral β-blocker users after cardiac and/or thoracic aortic surgery [7]. Despite differences in use, the indications of TDPB for POAF cases have been expanded. Our previous study lacked detailed data on individual cases. The present study comprised an expanded investigation that included a larger sample and detailed data on additional parameters, such as operation time, preoperative ejection fraction, preoperative creatinine, patch application day, and other medications in individual cases. We aimed to describe the efficacy of TDPB in patients with POAF after open-heart surgery.

## Methods

This study comprised an expanded investigation that included a larger sample and detailed data on additional parameters, such as operation time, preoperative ejection fraction, preoperative creatinine, patch application day, and other medications in individual cases. We aimed to describe the efficacy of TDPB in patients with POAF after open-heart surgery.

### Patients and data collection

Among 384 adult patients who underwent open-heart surgery at Okamura Memorial Hospital between December 2013 and March 2017, we searched the pharmacy department’s database and extracted details of cases wherein TDPB was used after open-heart surgery. The respective medical records were extracted from the medical record system and retrospectively analyzed. Data obtained included age, sex, disease, comorbidities, operation type, preoperative ejection fraction, preoperative creatinine, operation time, use of medications, electrocardiogram history, final electrocardiogram at discharge, history of electrical defibrillation, use of landiolol, and the number of Bisono tape® patches used. The definition of POAF was new-onset AF sustained for over 30 s, detected by either continuous telemetry in the intensive care unit, standard 12-lead electrocardiogram, or implanted devices. This study was conducted in accordance with the principles of the Declaration of Helsinki. The Institutional Review Board of Okamura Memorial Hospital approved the use of patient data for this retrospective study (approval number: A020-003, approval date: December 21, 2020). The need to obtain informed consent was waived by the Institutional Review Board of Okamura Memorial Hospital because of the retrospective nature of this study.

### Protocols

**Fig. 2:**
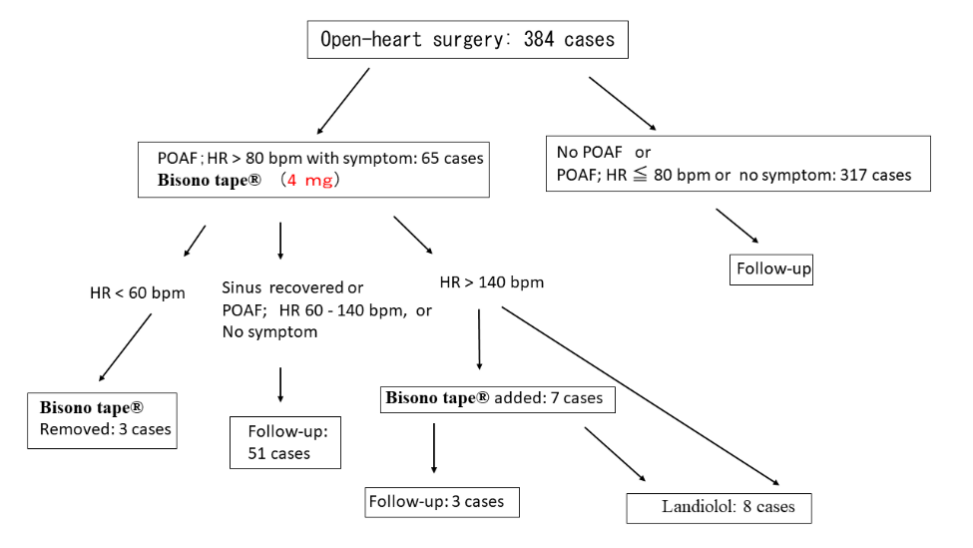
Protocol for postoperative atrial fibrillation. One Bisono tape® patch (4 mg) is applied for symptomatic patients with a HR of ≥ 80 bpm. The patch is removed if the HR drops below 60 bpm. If POAF occurs again, the patch is reapplied. If the HR remains at > 140 bpm, another patch is added. If the above does not yield favorable results and symptoms persist, a continuous infusion of landiolol should be administered. HR: heart rate; bpm: beats per minute; POAF: postoperative atrial fibrillation

The prophylaxis and anticoagulation procedures for POAF at our institution were as follows: oral β1 blocker (bisoprolol; 1.25 mg) was administered twice daily starting from the 1st day after open-heart surgery. The dose was increased to 2.5 mg twice daily starting from day 2, but not in cases of bradycardia with a heart rate (HR) of < 60 bpm. Patients with a low risk of postoperative rebleeding (determined on a case-by-case basis by the attending physician) were treated with subcutaneous heparin calcium starting from the second postoperative day, with 5,000 units twice daily for 5 days. In patients receiving warfarin, heparin calcium was discontinued when the prothrombin time-international normalized ratio was > 1.5. In cases, in which POAF developed and the patient was symptomatic despite the above treatments, a Bisono tape® transdermal patch was applied. Our protocol is shown in Fig. 2. One patch (4 mg) was applied when the HR was > 80 bpm, and symptoms, such as palpitations and hypotension, were present. If the HR decreased to < 60 bpm, the patch was removed and reapplied if POAF recurred. If the HR remained > 140 bpm, an additional patch was applied. If the above did not induce a response and the patient remained symptomatic, a continuous infusion of landiolol was initiated.

### Statistical analysis

Data are expressed as means ± standard deviations (minimum-maximum). Median values were calculated using Microsoft Excel 2019, version 2005 (Microsoft, Redmond, WA).

## Results

Sixty-seven of the 384 patients received TDPB. Two patients were excluded; in one the patch was used for HR control after a spontaneous return to normal sinus rhythm, and in the other, it was used for blood pressure control. The remaining 65 patients (16.9%) were included in the study analysis. Details of cases using the TDPB are shown in Table 1. The mean age was 72.7 ± 10.7 (range, 53–93) years. There were 30 men and 35 women included. Regarding the operation type, there were 51 cases of valve-related surgery, including 15 cases of maze procedure or pulmonary vein isolation surgery, six cases of coronary artery bypass surgery alone, two cases of Bentall surgery, five cases of thoracic aortic aneurysm resection, and one case of ventricular septal perforation occlusion. The average number of Bisono tape® prescriptions during hospitalization was 1.8 ± 1.1 (range, 1–5). In some cases, the tape was used daily because the patient remained in AF, while in others, one tape was prescribed for every POAF. The median first prescription date was postoperative day (POD) 2 (range, POD 0–37). In addition, 18 out of 65 patients (27.7%) recovered sinus rhythm within 24 h. The treatment of eight patients (12.3%) was switched to continuous infusion of landiolol due to tachycardia. In four cases, the patient’s treatment was switched to an infusion of landiolol without additional TDPB. Fifteen patients experienced persistent AF and were treated with electrical cardioversion during hospitalization. There were no serious complications (prolonged severe bradycardia, shock, etc.) that could be directly attributed to Bisono tape®. The TDPB was withdrawn from three patients with severe bradycardia (HR 30–40 bpm) early in this study. These patients subsequently recovered. One of the patients also simultaneously received a continuous infusion of low-dose dopamine. Five patients were discharged with AF. Despite using the POAF protocol, there were 15 cases, in which other drugs (digitalis, amiodaron, and verapamil around TDPB use) were used based on the judgment of the attending physician or the doctor on duty regarding the condition of the patient. One patient had a pacemaker implanted because of persistent sick sinus syndrome. There were three deaths, which were deemed unrelated to the use of TDPB.

**Table 1:** Details of cases in which transdermal patches of bisoprolol were used for postoperative atrial fibrillation

Age range (years)	Disease	Comorbidities	Operation Type	Op time (min)	EF (pre-op)	Pre-op Cr	Patch applied (POD)	No. of pieces used	Medications	24 h sinus	Final ECG	Complication
80–89	MR, TR, PAF	HT, DL	MAP, TAP, PVI	256	68.9	0.6	4	1		+	Sinus	
70–79	IHD	HT, DL, DM, obe.	OPCAB	227	61.6	1	1	1			Sinus	
50–59	IHD	obe.	OPCAB	334	42.4	0.8	1	1			Sinus	
60–69	MR, AF	HT, DL, obe.	MAP, MVP, maze	245	64.8	1.5	1	1			AF	DC removal
80–89	VSP	HT, DL, DM	VSP repair	277	44.0	0.9	2	1			Sinus	Death (2 POD)
80–89	ASR, IHD	HT	AVR, CABG	272	56.0	0.8	0	1			Sinus	
70–79	MR, PAF		MVR, maze	324	60.4	1.1	7	1		+	Sinus	
70–79	IHD	HT, DL, DM	MAP, CABG	393	38.7	1.5	2	1			Sinus	
80–89	IHD	HT, DM, HD	AVR, CABG	273	64.9	8.5	8(2), 24	3			Sinus	
70–79	DAA	HT	TAR	415	61.9	0.5	2, 5, 7	3	Verapamil		Sinus	
70–79	AS	HT, DM	AVR	196	51.0	0.6	7, 8	2			Sinus	
70–79	AS	DL	AVR	221	66.0	0.6	3	1		+	Sinus	
70–79	AS, OMI	HT, DM, HD	AVR	244	38.3	8.5	8	1		+	Sinus	
70–79	AR	HT	AVR (re-do)	333	59.4	1.31	3 (2)	2	Landiolol	+	Sinus	DC
80–89	MR, TR, PAF		MVP, MAP, TAP, maze	251	63.7	0.5	3	1		+	Sinus	
70–79	AR, MR, TR, PAF	HT, DL, obe.	AVR, MAP, maze	288	39.4	3.8	3	1			Sinus	
60–69	IHD	HT, DL, DM, obe	OPCAB	301	50.0	1.2	1	1		+	Sinus	
50–59	TAA	HT	Desc. A repl.	562	36.5	0.7	2 (2), 3, 4	4	Landiolol Digitalis		Sinus	DC
50–59	AAE, AR	HT, DL, obe.	Bentall	338	48.4	0.72	2	1		+	Sinus	
80–89	IHD, MR		MAP, CABG	357	19.4	1.11	8	1			Sinus	Removal
80–89	AS	HT	AVR	198	60.4	0.78	2 (2)	2	Landiolol Digitalis Verapamil		Sinus	
60–69	AS	HT, DL, DM	AVR	219	64.8	0.94	0	1	Landiolol		Sinus	
50–59	MR, TR, AF		MAP, TAP, maze	281	35.9	0.82	0,7	2			AF	RR7→DOA removal
70–79	AS, MR, AF	DL, obe.	AVR, MVP, maze	244	52.9	0.81	3	1			Sinus	DC
60–69	ASR, PAF	obe.	AVR, maze	239	34.7	1.51	10	1	Verapamil		Sinus	DC
60–69	IHD, AR	HT, DL, DM	AVR, CABG	377	27.4	1.04	3,4	2	Digitalis	+	Sinus	
30–39	MR		MAP, MVP	243	66.3	0.82	3	1		+	Sinus	
80–89	RV perforation	HT	CABG (emerg) RV repair	180	63.5	0.86	4,5	2			u/i	CI, death (30 POD)
70–79	AR		AVR	231	39.1	1	1,2,4(2)	4			Sinus	
60–69	IHD	HT, obe., HD	CABG	269	23.4	10.6	3,5,7	3			Sinus	
90–99	ASR	HT, DL	AVR	240	61.5	1	2,7,9	3			AF	DC
80–89	AS	HT	AVR	197	63.0	1.44	1	1			Sinus	
60–69	MR, TR	HT, obe., HD	MAP, MVP, TAP	210	57.8	0.47	8	1	Amiodaron	+	Sinus	
80–89	IHD, AS	HT, DL, obe.	AVR, CABG	334	60.8	0.7	1	1	Amiodaron	+	Sinus	
70–79	IHD, AS	HT, obe., HD	AVR, CABG	275	65.9	1	2,3,4, 5,6	5			Sinus	
70–79	MR, TR, OMI	HT, DL	MAP, TAP, CABG, maze	334	34.0	1.4	5,8	2			Sinus	DC
60–69	MR, TR	HT, obe., HD	MVR, TAP	435	62.6	0.7	6,7,8, 10,13	5	Amiodaron		Sinus	Temp. pacing
60–69	AR, MR, TR, AF	HT	AVR, MAP, TAP, maze	303	30.0	1.1	7	1			Sinus	DC
70–79	MR, TR	H, DM, obe.	MVR, TAP	354	61.5	0.8	3	1			Sinus	Death (1 m)
80–89	IHD	HT, DL, DM	CABG	280	42.0	2.7	4	1	Landiolol amiodaron		Sinus	
70–79	AR, AAE	HT, DL	AVR, Asc Ao repl.	350	44.8	0.93	2,5,8	3	Amiodaron		Sinus	DC
60–69	MR, TR, AF		MVR, TAP, maze	246	48.9	0.75	0	1			Sinus	
70–79	AR	HT	AVR (re-do)	333	59.4	1.31	2(2)	2	Landiolol		Sinus	DC
50–59	AS, Asc. AA		AVR, Asc Ao repl.	305	54.6	0.84	0	1			Sinus	DC
70–79	IHD	HT, DM, obe.	AVR, CABG, myectomy	297	62.3	0.7	2,6	2			Sinus	
50–59	AAE, AR	HT, DL, obe.	Bentall	338	48.4	0.72	2	1	Landiolol		Sinus	
70–79	TAAA	HT, DL, DM	TAAA repl.	408	58.3	1.39	2	1	Landiolol		Sinus	
70–79	MR, TR, AF		MVR, TAP, maze	251	57.0	1.11	17,20	2			Sinus	DC
80–89	ASR	Obe	AVR	193	65.5	0.58	1	1		+	Sinus	
70–79	AS	HT, DL	AVR	217	60.7	0.6	37	1			Sinus	
80–89	AS	HT, DL	AVR	227	61.7	0.7	5	1		+	Sinus	
80–89	AS	HT, DL	AVR	229	56.1	1.14	2,3,4, 6,11	5			Pacing	PM (SSS)
60–69	MSR, TR, AF	HT, DL, DM	MVR, TAP, maze	219	54.9	1.93	5	1			Sinus	DC
80–89	AS	obe.	AVR (re-do)	298	49.8	0.78	1, 5,7, 12	4			AF	
70–79	AS	HT, DL, obe.	AVR	167	50.2	0.84	2	1			Sinus	
70–79	MR, TR, IHD	HT, DL	MVP, MAP, TAP, CABG	311	57.7	1.27	1	1		+	Sinus	
80–89	AS	HT	AVR	162	52.3	1.07	1,3	2		+	Sinus	
60–69	MS, AS, TR	DL, obe.	DVR, maze	267	58.2	1.17	3(2),4	3	Digitalis Amiodaron		Sinus	DC
60–69	TAA	HT	Total arch	426	63.4	0.99	1	1		+	Sinus	
50–59	IHD, AR	HT, DL, obe.	AVR, CABG	361	58.5	0.99	8,9	2	Verapamil		Sinus	DC
60–69	AS		AVR	168	62.5	0.95	1	1			Sinus	
70–79	lt subcl. AA	HT, DL, DM	Total arch, omen.	390	60.0	0.47	1,2	2	Digitalis		Sinus	
70–79	AR, TR, PAF		AVR, TAP, PVI	228	45.8	0.84	10,17,18	3	Amiodaron Digitalis		AF	
90–99	AS, OMI		AVR	205	50.7	0.74	3	1			Sinus	
70–79	MR, HOCM	HT, DL	MVR, myectomy	226	61.8	0.52	12	1		+	Sinus	

The mean operative time was 286.5 ± 75.8 (range, 162–562) min. The mean preoperative EF was 53.2 ± 11.6% (range, 19.4–68.9%). The average number of Bisono tape prescriptions during hospitalization was 1.8 ± 1.1 (range, 1–5). The median first prescription date was on POD 2 (range, POD 0–37)

**Abbreviations**: AAE: aortic annular enlargement; AF: atrial fibrillation; AR: aortic valve regurgitation; AS: aortic valve stenosis; Asc, AA: ascending Asc. Ao: ASR: aortic stenosis and regurgitation; AVR: aortic valve replacement; CI: cerebral infarction; CABG: coronary artery bypass grafting; Cr: creatinine; DAA: dissecting aneurysm of the aorta; DM: diabetes mellitus; Desc. A: descending aorta; DC: direct current cardioversion; DL: dyslipidemia; DOA: dopamine; DVR: double valve replacement; ECG: electric cardiogram; EF: ejection fraction; emerg.: emergent; HR; heart rate; HT: hypertension: HOCM: hypertrophic obstructive cardiomyopathy: IHD: ischemic heart disease; MAP: mitral annulus plasty; MVP: mitral valve plasty; MR: mitral valve regurgitation; MVR: mitral valve replacement; MS: mitral valve stenosis; lt. subcl. AA: subclavian arterial aneurysm; Obe: obesity; OMI: old myocardial infarction; omen: omental packing; Op: operation; OPCAB: off-pump coronary artery bypass; PM: pacemaker; PAF: paroxysmal atrial fibrillation; POAF: postoperative atrial fibrillation; POD: postoperative day; repl.: replacement; PVI: pulmonary vein isolation; RR: RR interval; RV: right ventricle, SSS: sick sinus syndrome; TAA: thoracic aortic aneurysm; TAAA: thoracoabdominal aortic aneurysm; TAP: tricuspid annulus plasty; TAR: total arch replacement; Temp.: temporary; TR: tricuspid valve regurgitation; u/i: unidentified; VSP: ventricular septal perforation

## Discussion

In this study, we tested the efficacy of the TDPB in patients with POAF after open-heart surgery. Of the 65 patients who developed POAF, landiolol use was avoided in 57 (87.7%) patients. TDPB could be implemented as a safe and easy-to-use initial treatment for POAF after open-heart surgery.

Bisono tape® was the first TDPB developed in Japan and the first transdermal absorption-type β1 blocker to be developed globally; it is characterized by its ability to maintain a more stable blood concentration with continuous use compared to oral drugs [8]. The TDPB was launched in 2013 in 4-mg and 8-mg dosage forms; currently, a 2-mg dosage form is also available. Initially, it was indicated only for hypertension, but an indication for AF has recently been added. The most common side effects observed include pruritus, dermatitis, and erythema at the application site as well as abnormal laboratory changes, such as increased levels of triglycerides and alanine aminotransferase (glutamic pyruvic transaminase). As the patch is simply applied to the skin, it can be used in patients who cannot take oral medications. Considering the characteristics of this patch formulation, we believe its use may help avoid the side effects of oral β-blockers, such as severe bradycardia and cardiac depression, when applied during POAF. Bisono tape® 4 mg is equivalent to 2.5 mg of bisoprolol tablets [9], and its blood concentration is stable when used continuously. Therefore, when Bisono tape® is used immediately after surgery as a prophylactic for POAF, the drug’s effect is gradually diminished and may be prolonged, even if the patch is removed due to severe bradycardia. However, single-use applications are relatively safe because of the diminished effect of the patch [4].

POAF after open-heart surgery affects approximately 15–60% of patients, often during POD 2–4, and its incidence decreases with decreasing inflammatory findings [10]. The AATS guidelines recommend HR control resulting in sinus rhythm. In other countries, esmolol is available as a β-blocker for the treatment of POAF. However, in Japan, the use of esmolol is limited to monitored and intraoperative use. Although short-acting landiolol infusion is useful for this purpose, it is complicated, difficult to specify the infusion rate, and expensive [3]. Amiodarone is also effective [11]; however, the blood concentration is not readily elevated by this drug, and concomitant use with β-blockers might likely induce bradycardia. In addition, the half-life after the withdrawal of long-term amiodarone treatment can be up to 100 days [12]; moreover, amiodarone is difficult to use because it enhances the effects of warfarin and causes serious side effects, such as pulmonary fibrosis and liver damage, although rarely. Calcium channel blockers are recommended only when β-blockers are not available because of their strong cardiac depression. Furthermore, sodium channel blockers can cause ventricular tachycardia due to QT prolongation, and digitalis is not recommended due to problems, such as toxicity.

Among the 384 patients who underwent open-heart surgery in our study, 65 (16.9%) developed POAF and were prescribed Bisono tape®. Eighteen of the 65 patients (27.7%) recovered to sinus rhythm within 24 h after application of the drug. Although this was not the only effect of the drug, the concomitant use of an oral β-blocker made the treatment effective. We were unable to investigate HR control in detail; however, we observed a gradual reduction in HR after patch application, as previously reported [6, 13], which may have contributed to the reduction in symptoms. Bisono tape® is better tolerated than a continuous infusion of landiolol and does not interfere with patients’ activities of daily living. At a price of ¥86.6 (0.8 USD) per 4 mg, Bisono tape® is also less expensive than landiolol infusion (¥6,577 [63 USD] per 50 mg). In this study, landiolol was required in eight (12.3%) out of 65 patients. As POAF can occur at any time of the day or night, it is very labor-intensive for medical staff to respond to each incident. The protocol we developed is straightforward and helps reduce the workload of medical staff.

We hope that the same POAF protocol will also be useful in outpatients who may have paroxysmal AF. Wearable wristwatches could be used to diagnose paroxysmal AF [14] and may allow HR estimation to prompt patch removal or initiate automatic hospital calls.

This study had several limitations. First, this was a single-center retrospective study involving a small sample size, which may have led to selection bias. Second, we did not include a control group. Third, the effect of HR control from POAF resulted from oral bisoprolol and other arrhythmic drugs in addition to the TDPB; therefore, no simple conclusions, such as the effect of the patch alone or whether the use of the patch can help avoid the side effects of oral beta-blockers, can be drawn. Thus, a prospective randomized controlled study is required to validate the safety and efficacy of the TDPB in patients with POAF.

### Conclusions

We examined the efficacy of a bisoprolol transdermal patch for managing POAF and flutter in thoracic surgical procedures. We found that single-use TDPB may be an option for HR control during the treatment of POAF after open-heart surgery.

## Data Availability

All data generated or analyzed during this study are included in this published article.

## List of Abbreviations

AATS: American Association for Thoracic Surgery
HR: Heart rate
POAF: Postoperative atrial fibrillation
POD: Postoperative day
